# Turner Syndrome With Isochromosome Structural Abnormalities: A Case Report

**DOI:** 10.7759/cureus.40516

**Published:** 2023-06-16

**Authors:** Tahmina Ferdousi, Hurjahan Banu, Nusrat Sultana, Hafsa Mahrukh, Muhammad Abul Hasanat

**Affiliations:** 1 Department of Endocrinology, Bangabandhu Sheikh Mujib Medical University, Dhaka, BGD

**Keywords:** short stature, mosaic turner syndrome, amenorrhea, isochromosome x, turner syndrome

## Abstract

Turner syndrome (TS) is the most common cause of short stature and delayed puberty in females. Approximately half of the patients have the classic form with a genotype of 45,XO, one-fourth of patients have different mosaic forms, and the remaining one-fourth have structural abnormalities on the X chromosome. Among the structural abnormalities, the most common is isochromosome Xq. Females with structural variants of TS can present with delayed menarche, amenorrhea, and infertility rather than classic manifestations of TS.

This study describes two rare variants of TS. One was a structural abnormality on the X chromosome, 46X,iso(Xq), and the other involves a mosaic variety of TS, including isochromosome X in the form of 45,XO/46X,iso(Xq). Both patients presented with short stature and secondary amenorrhea without classic manifestations of TS.

In TS with or without mosaicism, the frequency of isochromosomes is reported to be about 15% to 18%. Owing to the absence of classical manifestations of TS, diagnosis may be delayed or missed. Therefore, females of short stature with secondary amenorrhea should be evaluated for rare variants of TS by chromosomal analysis.

## Introduction

Turner syndrome (TS), which was first described by Henry Turner in 1938, is a female genetic disorder in which the X chromosome can be completely or partially absent [1]. With an estimated frequency of 1/2,500, TS is one of the most prevalent chromosomal abnormalities in live female births [2]. Approximately 50% of TS patients have the classic form of 45,XO, 25% of cases involve mosaic forms, and the remaining cases include structural abnormalities of the X chromosome [3]. Isochromosome Xq is the most prevalent among the structural X chromosome abnormalities [4]. TS with a 45,XO karyotype has been reported to occur in 1% to 2% of human conceptions, 10% of first-trimester pregnancy losses, and 1% of stillbirths. By the 28th week of gestation, more than 99% of 45,XO fetuses are aborted, suggesting that surviving 45,XO individuals must have mosaicism for a different cell line [5,6]. TS typically presents as short stature paired with other characteristics such as low-set ears, webbed neck, and skeletal deformities. Systemic abnormalities, including gonadal dysgenesis, renal disease, heart disease, and hearing impairment, are commonly present [7]. Oedema, facial dysmorphism, and other severe defects typically lead to TS diagnosis at birth. However, mild cases due to variant forms may only become apparent later in adulthood, with characteristic symptoms, including infertility, amenorrhea, and delayed menarche [8]. This paper describes two cases of variant TS, one caused by isochromosome Xq and the other identified as isochromosome mosaic Turner - 45,XO/46X,iso(Xq).

## Case presentation

Case 1

A 25-year-old woman attended our Department of Endocrinology for an evaluation of her short stature and secondary amenorrhea. She had a history of three episodes of scanty withdrawal bleeding following the use of combined oral contraceptives. Aside from the menstrual issues, she had no significant medical issues. She was the first child of nonconsanguineous parents, and her antenatal and postnatal periods were uneventful. There was no known inherited or familial illness, short stature, or delayed menarche among female family members. Her intelligence was normal. On examination, her height was 132 cm (<5th percentile), weight was 52 kg, upper segment (US) was 64 cm, lower segment (LS) was 68 cm, and US:LS was 0.94. Her height age was nine years, and her weight age was 15 years. The computed mid-parental height was 146 cm. She was obese at 29.84 kg/m^2^ body mass index and had acanthosis nigricans. She had no phenotypical abnormalities other than the wide-carrying angle of the elbows. Tanner staging was B3, P4, and she had normal female genitalia. Her vitals were normal. Laboratory testing revealed metabolic dysfunction in the form of type 2 diabetes mellitus (T2DM), dyslipidemia, and raised alanine transaminase and hypergonadotropic hypogonadism with normal thyroid function test and prolactin level (Table 1).

**Table 1:** Metabolic and hormonal profile of the two cases. *According to ADA standards of diabetes care 2023. **During the follicular phase of the menstrual cycle. ADA, American Diabetes Association; ALT, alanine transaminase; DM, diabetes mellitus; HDL, high-density lipoprotein; LDL, low-density lipoprotein; FSH, follicle-stimulating hormone; LH, luteinizing hormone; TSH, thyroid-stimulating hormone; FT4, free thyroxine

Name of the investigations	Case 1	Case 2	Reference value
Fasting plasma glucose	9.7	5.3	Normal: 3.9-5.6 mmol/L*; prediabetes: 5.6-6.9 mmol/L*; DM: ≥7 mmol/L*
2 hours after 75 gm glucose	13.6	6.5	Normal: <7.8 mmol/L*; prediabetes: 7.8-11.0 mmol/L*; DM: ≥11.1 mmol/L*
ALT	76	15	0-55 U/L
Total cholesterol	302	180	<200 mg/dL
HDL cholesterol	53	50	>40 mg/dL
LDL cholesterol	219	97	<130 mg/dL
Triglycerides	152	148	<150 mg/dL
FSH	63.23	60.02	3-20 IU/L**
LH	17.29	9.647	5-15 IU/L**
Serum estradiol	10.5	13.2	21-251 pg/mL**
Serum testosterone	0.49	0.17	0.05-1.20 ng/mL
TSH	4.46	3.35	0.55-4.78 mIU/L
FT4	12.20	18.89	9.0-19.05 pmol/L
Prolactin	7.07	7.24	1.8-20.3 ng/mL

**Figure 1:**
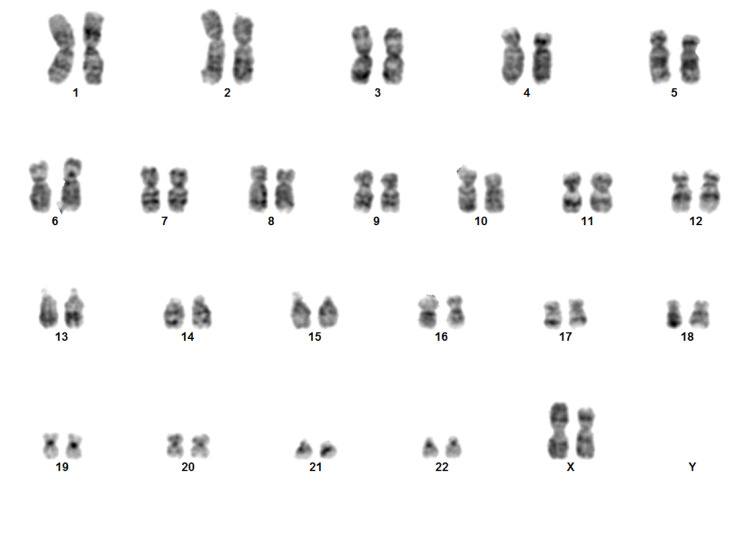
Karyotype of the first case showing 46X,iso(Xq).

Transabdominal ultrasound showed a small uterus with thin endometrium and myometrium, measuring about 50 mm × 12 mm × 21 mm, a volume of 6.8 cm^3^, endometrial thickness of 3.7 mm, and ovary-like structures in both adnexal regions with volumes of 0.45 cm^3^ (right) and 0.16 cm^3^ (left). Bone mineral density testing showed osteoporotic changes (*Z*-score, −2.5) in the lumbar vertebrae. Her serum calcium was within the normal range (9.2 mg/dL), and there was mild vitamin D deficiency (15.72 ng/mL). Finally, chromosomal analysis was performed by peripheral blood lymphocyte culture, which showed 46X,iso(Xq) (Figure 1). The patient was managed with conjugated estrogen therapy (0.625 mcg), oral antidiabetic drug (metformin XR 500 mg), and lipid-lowering agents (atorvastatin 10 mg) along with lifestyle modification. After six months of follow-up, menstruation had not started, and ultrasonography was advised, but the patient denied to do.

Case 2

**Figure 2:**
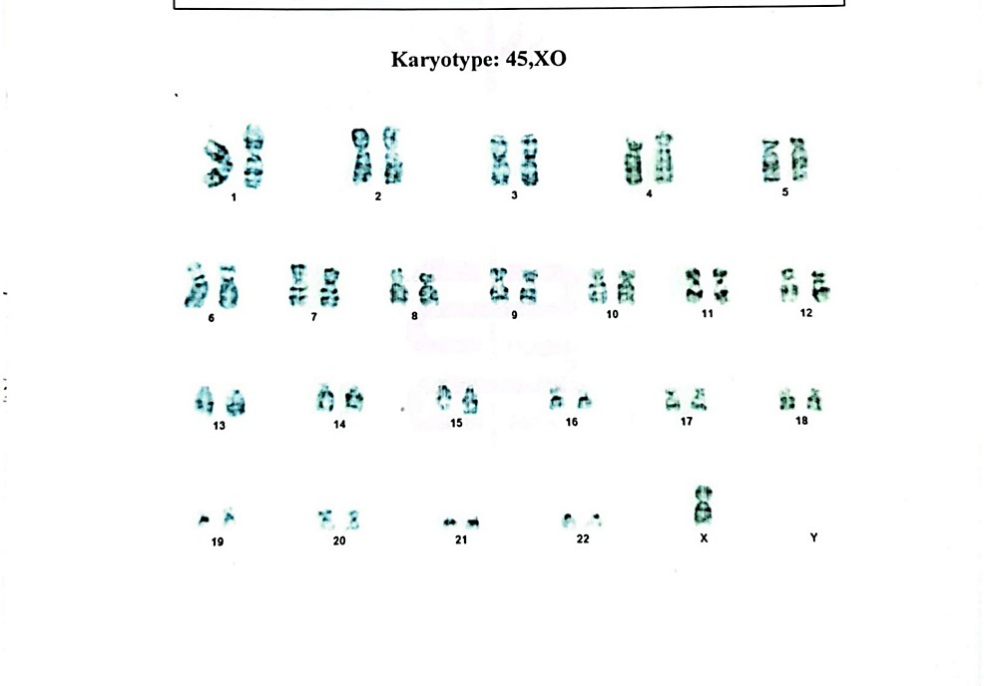
Karyotyping of the second case showing 45,XO.

**Figure 3:**
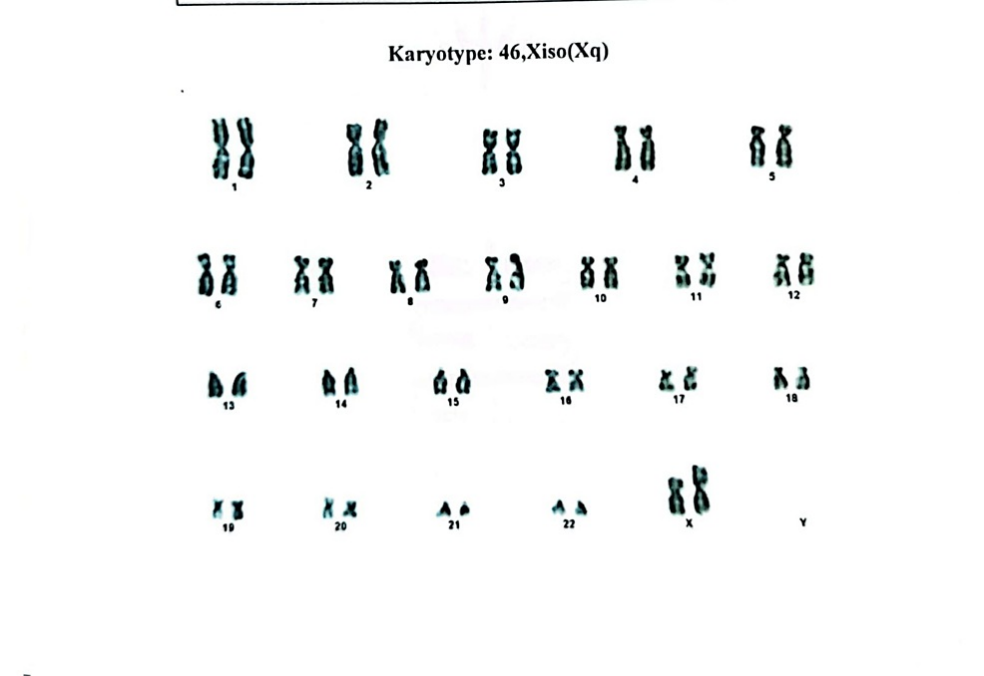
Karyotyping of the second case showing 46X,iso(Xq).

Our second case involved a 17-year-old girl who attended a consultation for evaluation of her short stature and secondary amenorrhea. Her menstruation spontaneously started when she was 12 years old and ceased after three or four cycles. She was the second child of nonconsanguineous parents, and she had uneventful antenatal, natal, and postnatal periods. There was no family history of short stature or delay in menses of female family members. Her intelligence was normal. On examination, her height was 138 cm (<5th percentile) and her weight was 53 kg. Her BMI was 27.83 kg/m^2^ at the 92nd percentile, which falls in the overweight category. Her height age was 10 years, and her weightage was 15.5 years. The computed mid-parental height was 150.5 cm. Except for low-set ears, she did not have a typical TS phenotype. Tanner staging revealed B4 and P3. The patient’s vitals were normal. Her routine investigations were unremarkable, but hormonal evaluation showed hypergonadotropic hypogonadism with normal testosterone, thyroid function test, and prolactin level (Table 1). Transabdominal ultrasound showed a small uterus with thin endometrium and myometrium, measuring about 4.0 cm × 1.8 cm, and both adnexal regions were normal with 1.4 cm (right ovary) and 1.7 cm (left ovary). Bone mineral density testing showed a normal bone mass (*Z-*score, −1.8) of the lumbar vertebrae. Her chromosomal analysis was performed by peripheral blood lymphocyte culture, which showed mosaicism 45,XO/46X,iso(Xq)(2:1) (Figures 2-3). The patient was treated with conjugated estrogen (0.625 mcg). Follow-up ultrasonography after six months showed the increasing size of the uterus measuring about 6.1 cm × 3.2 cm × 1.9 cm.

 

## Discussion

This study features two patients with TS, a 25-year-old woman and a 17-year-old girl, who did not exhibit classic TS manifestations. Karyotypes of the 25- and 17-year-old patients were 46X,iso(Xq) and 45,XO/46X,iso(Xq)(2:1), respectively. The first patient had an X chromosome structural abnormality, and the second had a mosaic of TS, including X chromosome structural abnormalities. There are numerous types of TS with structural abnormalities of the X chromosome, including deletion (Xp or Xq), isochromosome X [46X,i(X)], and ring chromosome [46X,r(X)] [9]. In addition, TS varieties with mosaicism include 45,XO/46XX, 45,XO/46XY, and 45,XO/47XXX [9]. The most common structural abnormality of the X chromosome is 46X,i(Xq). An isochromosome is a structural chromosomal aberration formed by the centromeric fusion of two copies of short arms or long arms. One study found that TS cases with or without mosaicism had an isochromosome frequency of 15% to 18% [10]. The most typical clinical feature of TS is short stature, which can be explained by short-stature homeobox (SHOX) haploinsufficiency [11]. In the 46X,i(Xq) karyotype, which has isochromosome on the long arms, short stature is the common feature. On the other hand, 46X,i(Xp), which has an isochromosome on the short arm of the X chromosome and is a very rare entity, gonadal dysfunction is more likely to occur. Our two cases presented with short stature (<5th percentile) and secondary amenorrhea. In such cases, the diagnosis of variant TS can be delayed because classic features of TS can be absent [12]. Our two patients also received a diagnosis at a late age, 25 and 17 years, respectively. Among the mosaic variants of TS, the most common form of mosaicism is 45,XO/46,XX (15%) [13], while 45,XO/46X,iso(Xq) mosaicism is a rare entity (8%-9%) [12,14], which includes both the cell line with the pathological cytogenetic structure and structural abnormality of the normal X chromosome. These patients with mosaic TS may have a near-normal phenotype rather than typical TS features. In 2021, a review highlighted that the mean adult height of classic TS is an average of 20 cm shorter than the general female population in untreated patients [15]. The probability of somatic anomaly is lower, and the mean adult height is higher in mosaic females [9]. About 3% of the 45,XO females and up to 20% of mosaic females have spontaneous menstruation [9]. The frequency of impaired glucose metabolism varies with karyotype; more women with mosaic TS have normal glucose tolerance compared with women with 45,XO. In studies, T2DM was found in 10% of women with TS [16]; the proportion was 18% in women with 45,XO, 23% in women with an Xp deletion, and 43% in women with isochromosome Xq [17]. These findings suggest that extra copies of Xq significantly increase the risk of developing T2DM. Our first case of isochromosome Xq had a T2DM diagnosis based on abnormal oral glucose tolerance test reports. In addition, a meta­-analysis in 2018 found the overall prevalence of autoimmune thyroid diseases in women with TS to be 38.6%, which increases with age [18]. However, women with an isochromosome are more likely to develop autoimmune thyroid disorders and hypothyroidism [19]. In addition, relative to individuals with mosaic karyotypes, people with the 45,XO or 46X,i(Xq) karyotype have a higher chance of hearing loss. Aging and hearing loss were observed to be directly proportional [20]. Both of our patients had normal thyroid function and no hearing loss at diagnosis and follow-up six months later; however, they may be at increased risk of hearing loss at later ages.

## Conclusions

We described two uncommon variants of TS: both patients presented with short stature, secondary amenorrhea, and the absence of other classical manifestations of TS. It is recommended that any secondary amenorrheic female with short stature should be tested for TS using chromosomal analysis at any stage of her reproductive life. Further research on variant TS is needed to better understand this complex genetic disorder.

## References

[1] Turner HH (1938). A syndrome of infantilism, congenital webbed neck, and cubitus valgus. Endocrinology.

[2] Nielsen J, Wohlert M (1991). Chromosome abnormalities found among 34,910 newborn children: results from a 13-year incidence study in Arhus, Denmark. Hum Genet.

[3] Gravholt CH (2005). Epidemiological, endocrine and metabolic features in Turner syndrome. Arq Bras Endocrinol Metabol.

[4] Al Alwan I, Khadora M, Amir 1st (2014). Turner syndrome genotype and phenotype and their effect on presenting features and timing of Diagnosis. Int J Health Sci (Qassim).

[5] Hook EB, Warburton D (1983). The distribution of chromosomal genotypes associated with Turner's syndrome: livebirth prevalence rates and evidence for diminished fetal mortality and severity in genotypes associated with structural X abnormalities or mosaicism. Hum Genet.

[6] Held KR, Kerber S, Kaminsky E, Singh S, Goetz P, Seemanova E, Goedde HW (1992). Mosaicism in 45,X Turner syndrome: does survival in early pregnancy depend on the presence of two sex chromosomes?. Hum Genet.

[7] Oliveira RM, Verreschi IT, Lipay MV, Eça LP, Guedes AD, Bianco B (2009). Y chromosome in Turner syndrome: review of the literature. Sao Paulo Med J.

[8] Rosenfeld RG (2000). Turner's syndrome: a growing concern. J Pediatr.

[9] Gürsoy S, Erçal D (2017). Turner syndrome and its variants. J Pediatr Res.

[10] Wolff DJ, Van Dyke DL, Powell CM (2010). Laboratory guideline for Turner syndrome. Genet Med.

[11] Cuesta Hernández M, Rueda Valencia ME, Pérez Rodríguez O, López de Lara D (2015). X isochromosomes: delayed diagnosis of Turner's syndrome. An Pediatr (Barc).

[12] Sybert VP, McCauley E (2004). Turner's syndrome. N Engl J Med.

[13] Rapaport R (2008). Hypofunction of the Ovaries. Nelson Textbook of Pediatrics, 18th ed.

[14] Akbaş E, Yazıcı FG, Durukan H, Topal H, Erdoğan NE (2014). Cytogenetic and clinical evaluation of two cases that have 45,X/46,X,i(Xq) and 46,X,i(Xq) karyotype 45,X/46,X,i(Xq) ve 46,X,i(Xq). J Clin Exp Invest.

[15] Dantas NC, Braz AF, Malaquias A (2021). Adult height in 299 patients with Turner syndrome with or without growth hormone therapy: results and literature review. Horm Res Paediatr.

[16] Gravholt CH, Viuff MH, Brun S, Stochholm K, Andersen NH (2019). Turner syndrome: mechanisms and management. Nat Rev Endocrinol.

[17] Bakalov VK, Cheng C, Zhou J, Bondy CA (2009). X-chromosome gene dosage and the risk of diabetes in Turner syndrome. J Clin Endocrinol Metab.

[18] Mohamed SO, Elkhidir IH, Abuzied AI, Noureddin AA, Ibrahim GA, Mahmoud AA (2018). Prevalence of autoimmune thyroid diseases among the Turner syndrome patients: meta-analysis of cross sectional studies. BMC Res Notes.

[19] Libert C, Dejager L, Pinheiro I (2010). The X chromosome in immune functions: when a chromosome makes the difference. Nat Rev Immunol.

[20] Oliveira CS, Ribeiro FM, Lago R, Alves C (2013). Audiological abnormalities in patients with Turner syndrome. Am J Audiol.

